# Prediction of size-resolved number concentration of cloud condensation nuclei and long-term measurements of their activation characteristics

**DOI:** 10.1038/s41598-017-05998-3

**Published:** 2017-07-19

**Authors:** H. C. Che, X. Y. Zhang, L. Zhang, Y. Q. Wang, Y. M. Zhang, X. J. Shen, Q. L. Ma, J. Y. Sun, J. T. Zhong

**Affiliations:** 1 Key Laboratory of Atmospheric Chemistry of CMA, Institute of Atmospheric Composition, Chinese Academy of Meteorological Sciences, Beijing, 100081 China; 2 College of Earth Science, University of Chinese Academy of Sciences, Beijing, 100049 China; 3 State Key Laboratory of Severe Weather, Chinese Academy of Meteorological Sciences, Beijing, 100081 China; 4 Center for Excellence in Regional Atmospheric Environment, IUN, CAS, Xiamen, 361021 China; 5 LinAn Regional Global Atmosphere Watch Station, LinAn, 311307 China; 6 State Key Laboratory of Cryospheric Sciences, Cold and Arid Region Environmental and Engineering Research Institute, Chinese Academy of Sciences, Lanzhou, 730000 China

## Abstract

Atmospheric aerosol particles acting as cloud condensation nuclei (CCN) are key elements in the hydrological cycle and climate. To improve our understanding of the activation characteristics of CCN and to obtain accurate predictions of their concentrations, a long-term field campaign was carried out in the Yangtze River Delta, China. The results indicated that the CCN were easier to activate in this relatively polluted rural station than in clean (e.g., the Amazon region) or dusty (e.g., Kanpur-spring) locations, but were harder to activate than in more polluted urban areas (e.g., Beijing). An improved method, using two additional parameters—the maximum activation fraction and the degree of heterogeneity, is proposed to predict the accurate, size-resolved concentration of CCN. The value ranges and prediction uncertainties of these parameters were evaluated. The CCN predicted using this improved method with size-resolved chemical compositions under an assumption that all particles were internally mixed showed the best agreement with the long-term field measurements.

## Introduction

Cloud condensation nuclei (CCN) are aerosol particles that enable the formation of cloud droplets at a given level of supersaturation. The distribution and characteristics of CCN influence the microphysical properties of the cloud, cloud lifetime, precipitation, hydrological cycle, and therefore the climate^1–4^. The greatest challenges in our current understanding of climate change are associated with the large uncertainties in the spatial and temporal distribution of CCN and their interactions with clouds^1,5,6^. Measurements and studies of CCN closure should be performed to reduce these uncertainties. Measurements of CCN can help us to better understand the variation of their concentration and activation characteristics, and closure studies can be used to test our knowledge of the controlling physical and chemical factors, to determine the best prediction methods for CCN, and to reduce the uncertainties caused by CCN.

Numerous field experiments^7–14^ and closure studies^11,14–17^ have been carried out worldwide to determine and analyze the characteristics of CCN. There is general agreement that the activation of CCN at a given level of supersaturation depends primarily on the particle size, followed by the chemical composition and mixing status; however, the relative importance of these characteristics may vary greatly in different environments and at different locations^7,11,18,19^. With respect to the prediction of the total concentration of CCN, good results have been achieved based on the *κ*-Kӧhler function^11,20,21^, where *κ* is the hygroscopicity of the particles and varies according to the chemical composition.

Most of these studies were performed in clean environments with short time durations (several weeks at most) and only total concentrations of CCN were predicted. As a result of the high concentration and complex chemical composition of aerosols in polluted environments, the concentration and activation characteristics of CCN in polluted areas may vary greatly from those under clean conditions^11,12^. In addition, the current method for the prediction of CCN generates a theoretically ideal shape for the number size distribution of CCN, which is not consistent with the real situation, especially at small particle diameters. An accurate prediction of the size-resolved concentration of CCN is needed under particular circumstances, such as new particle formation (NPF) events with high aerosol concentrations at small diameters^22^. The size-resolved concentration of CCN at high levels of supersaturation is also important because their critical diameters are fairly small. In addition, because the total CCN concentration is usually integrated from the size-resolved CCN concentration, an accurate prediction of the size distribution of the CCN concentration would also benefit the prediction of the total concentration. In polluted areas, the abundance of ultrafine aerosols means that the size distribution of CCN is important in their accurate prediction. The chemical composition of aerosols shows significant variations in polluted environments^23^, which may further influence the CCN.

China is one of the most polluted countries in the world and has extremely high concentrations of aerosols^24,25^. Previous measurements of CCN have mainly been carried out on the North China Plain and the Pearl River Delta region, mostly over short durations of time^11,15,19,26–28^. The Yangtze River Delta (YRD) region, which has long been an industrial heartland and prime economic region of China, suffers badly from haze pollution^29^. In 2013, the YRD region suffered from heavy haze pollution with a strong intensity, long duration, and extensive coverage^30^. Therefore, long-term measurements of the activation of CCN and accurate predictions of the concentration of CCN are needed in this area. NPF events frequently occur in the YRD and therefore the size-resolved concentration of CCN may also important in this region^31^.

A long-term campaign to measure the *in situ* CCN activation was launched at a regional GAW (Global Atmosphere Watch) station LinAn in the YRD from January to October 2013. The long-term activation characteristics of CCN were investigated and an improved method of predicting the size distribution of CCN is proposed with two additional parameters: the maximum activation fraction and the degree of heterogeneity. The range of these two new parameters and their prediction errors were investigated. Different prediction methods for the concentration of CCN were compared and analyzed.

## Results

### Mean CCN activation characteristics during the measurement campaign

**Figure 1:**
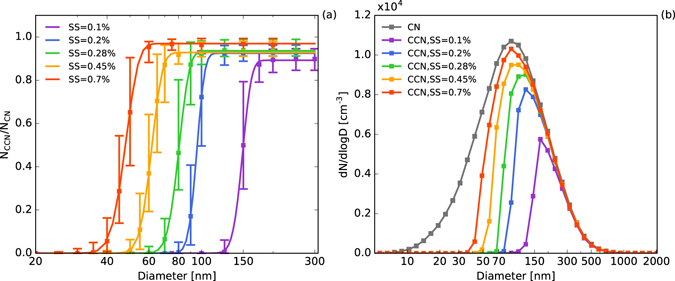
(**a**) Average CCN activation spectra and (**b**) average CCN and CN size distributions obtained during the measurement campaign at five different levels of supersaturation. The symbols represent the median value of all measured spectra and the lower and upper bars represent the quartile errors.

Figure 1 shows the average CCN activation spectra and number size distribution obtained during the campaign at five different supersaturations. By fitting with cumulative Gaussian distribution function (CDF), the following parameters were derived from the three-parameter CDF of each measured CCN efficiency spectrum^32^: the maximum activated fraction *MAF*_*f*_, the midpoint activation diameter *D*_*a*_, and the standard deviation *σ*_*a*_ of CDF fits; the following parameters were derived from two-parameter CDF by assuming *MAF*_*f*_ = 1: the midpoint activation diameter *D*_*t*_ and the standard deviation *σ*_*t*_ of the 2-parameter CDF fit (detailed in Methods section). The average activation parameters derived from the two CDFs are summarized in Table 1.

**Table 1:** Average CCN parameters obtained during the campaign determined from three- and two-parameter CDFs.

*SS* (%)	*D*_a_ (nm)	*D*_t_ (nm)	*MAF*_f_	*σ*_a_	*σ*_t_	*σ*_a_/*D*_a_	*σ*_t/_ *D*_t_
0.1	148.96 ± 9.29	152.13 ± 10.32	0.89 ± 0.07	9.46 ± 3.77	11.64 ± 3.99	0.06 ± 0.03	0.08 ± 0.03
0.2	95.88 ± 6.91	97.56 ± 7.79	0.92 ± 0.05	6.10 ± 3.96	7.72 ± 4.88	0.06 ± 0.04	0.08 ± 0.05
0.28	80.07 ± 5.72	81.22 ± 6.12	0.94 ± 0.04	6.06 ± 4.23	7.65 ± 4.73	0.08 ± 0.05	0.09 ± 0.05
0.45	61.84 ± 5.79	62.83 ± 5.96	0.93 ± 0.04	5.51 ± 3.69	6.91 ± 4.15	0.09 ± 0.06	0.11 ± 0.06
0.7	48.26 ± 4.97	48.90 ± 5.01	0.96 ± 0.03	5.34 ± 3.38	6.09 ± 3.70	0.11 ± 0.06	0.12 ± 0.07
***SS*** **(%)**	***κ***_**a**_	***κ***_**t**_	***N***_**CCN**_ **(cm** ^**−3**^ **)**		***N***_**CN**_ **(cm** ^**−3**^ **)**		***N***_**CCN**_ **/** ***N***_**CN**_
0.1	0.42 ± 0.08	0.40 ± 0.08	2252 ± 1367		10246 ± 4760		0.23 ± 0.12
0.2	0.40 ± 0.09	0.38 ± 0.09	4081 ± 2172		10593 ± 5319		0.41 ± 0.15
0.28	0.35 ± 0.08	0.33 ± 0.08	5003 ± 2492		10697 ± 5679		0.50 ± 0.16
0.45	0.30 ± 0.07	0.28 ± 0.07	6271 ± 2787		10270 ± 4796		0.62 ± 0.17
0.7	0.26 ± 0.07	0.25 ± 0.07	7116 ± 3162		10214 ± 4704		0.71 ± 0.16
All	0.34 ± 0.09	0.33 ± 0.09	—		10405 ± 5073		—

The data are presented as arithmetic mean ± standard deviation values.

Figure 1a and Table 1 show that the maximum active fraction (*MAF*_*f*_, derived from the 3-parameter CDF fitting, detailed in Methods) was close to unity at high levels of supersaturation. However, when *SS* = 0.1%, the *MAF*_*f*_ was about 0.89, indicating that about 10% of the aerosol particles in the size range 150–200 nm were inactive CCN, such as freshly emitted soot. The *MAF*_f_ at the LinAn sampling site was similar to that found in the Amazon rain forest^33^ and higher than the values recorded at polluted urban sites^11,34^, indicating the larger fraction of soot particles at urban sites. The average CCN number concentrations were calculated by multiplying the interpolated average CN size distribution by the average active fraction of the CCN. Figure 1b shows that the number size distributions of the CN and CCN were mono-modal. Almost all particles >300 nm were activated at the measured supersaturations and no CCN were observed for particles <30 nm, showing the effect of particle size on the activation of CCN.

Figure 1 and Table 1 show that the critical active diameters for CCN-active particles (*D*_a_, derived from the 3-parameter CDF fitting, detailed in Methods) and the total particles (*D*_t_, derived from the 3-parameter CDF fitting, detailed in Methods) varied from about 45 to 150 nm, with *SS* ranging from 0.7 to 0.1%. The ratios *σ*_a_/*D*_a_ and *σ*_t_/*D*_t_, i.e. the ratio of fitting standard deviation and critical active diameter, which represent the degree of heterogeneity of CCN-active particles around *D*_a_ and the overall degree of heterogeneity of CCN-active and -inactive particles around *D*_t_, respectively (detailed in Methods), decreased with increasing diameter of the particles, showing that the larger particles were more homogeneous and more internally mixed. At the same level of supersaturation, the critical active diameters at the LinAn site were far smaller than at Kanpur-spring^35^ and in the Amazon region^33^, larger than in Beijing^36^, and similar to those recorded in Guangzhou^32^. This is consistent with the fact that Kanpur-spring and the Amazon region are characterized by large fractions of mineral and organic aerosols, respectively, and Beijing has a larger fraction of inorganic particles. The ratios of *σ*_a_/*D*_a_ and *σ*_t_/*D*_t_ at LinAn were similar to those in the Amazon region, but smaller than at Kanpur-spring and Guangzhou, indicating that the aerosols in LinAn were more aged and more homogenous.

The campaign-averaged values for *κ*_a_ and *κ*_t_ represent the hygroscopicity of the CCN-active particles and the total particles (including both CCN-active and -inactive particles) around *D*_a_ and *D*_t_ respectively (detailed in Methods); both values increased with an increase in the critical active diameters, indicating that the larger particles were more hygroscopic. For smaller particles (about 40–50 nm), *κ*_a_ was almost the same as *κ*_t_, whereas for particles between 100 and 150 nm, *κ*_a_ > *κ*_t_, which may imply that smaller particles contain more hydrophobic components, whereas larger particles have a large fraction of hygroscopic components, such as inorganic species.

The peak CN concentration was at diameter of about 90 nm, with total particle number concentrations of about 10,405 cm^−3^, which was significantly lower than in urban areas of China^11,19^, but higher than in Korea, Japan, and North America^37–39^. This indicates the high aerosol loading present in China, even in rural areas, which could influence the formation and characteristics of cloud or fog. The CCN concentration at *SS* = 0.1% only accounted for about 20% of the total CN, with the peak concentration at about 200 nm. At higher values of supersaturation (0.28–0.7%), the size distribution of the CCN was close to that of the CN, with the total concentration of CCN increased from about 5000 to 7100 cm^−3^ and the concentration of CCN at *SS* = 0.7% accounting for about 70% of the total CN.

### Prediction of CCN based on long-term measurements

#### Effective hygroscopicity parameters and chemical composition of aerosols

The predictions of the CCN were modeled using the *κ*-Kӧhler theory (Eq. 7 in Methods) based on the measured particle number size distribution and the chemical composition. The *κ* value for a particle consisting of multiple components was determined using a simple Zdanovskii–Stokes–Robinso mixing rule^21^:

1 $$\kappa =\sum _{i}{\nu }_{i}{\kappa }_{i}$$

where the subscript *i* denotes the species and *ν*_*i*_ and *κ*_*i*_ are the volume fraction and corresponding hygroscopic capability of each species, respectively. However, the value of *κ* derived from Eq. 1 needs detailed information on the chemical components of the particle and is unlikely be applicable worldwide. Therefore, a simplified method was used based on the chemical composition determined by aerosol mass spectrometry (AMS)^33,40^:

2 $$\kappa ={\kappa }_{org}\times {f}_{org}+{\kappa }_{inorg}\times {f}_{inorg}\,$$

where *κ*_org_ and *κ*_inorg_ are the *κ* values for organic and inorganic aerosols, respectively, and *f*_org_ and *f*_inorg_ are the mass fractions of organic and inorganic aerosols, respectively.

The mass fractions were used here for first-order approximations^33,36,40^, assuming that the density of each component is similar to the overall particle density, which is reasonable for particles mainly composed of organics (∼1.3–1.4 g cm^−3^), sulfate (∼1.8 g cm^−3^) and nitrate (∼1.7 g cm^−3^)^20,41^. In previous studies in the Amazon region, Guangzhou, and Beijing^33,36,40^, values of 0.1 and 0.6 were used for *κ*_org_ and *κ*_inorg_; however, the values of *κ*_org_ and *κ*_inorg_ at the LinAn regional station may be different as a result of the different of chemical composition of the aerosols. Therefore, the *κ* analyses were performed based on size-resolved chemical composition measured by AMS.

**Figure 2:**
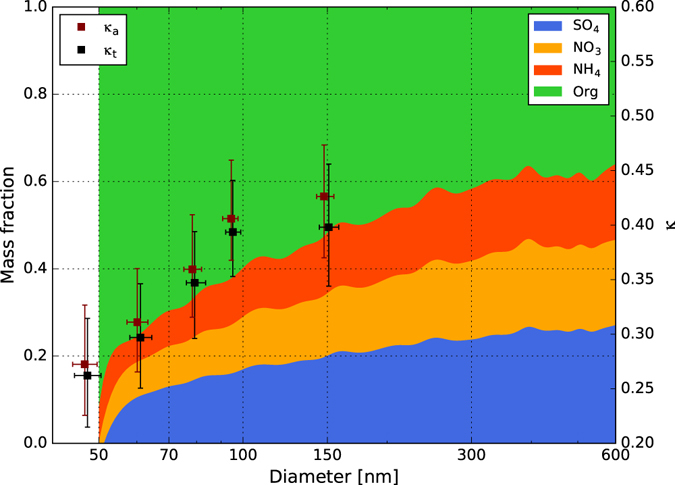
Average size distributions of the mass fractions of organic and inorganic compounds (colored areas), determined by AMS, plotted against the observed effective hygroscopicity parameters *κ*_a_ and *κ*_t_. The data points are median values measured at five different levels of supersaturation and the lower and upper bars represent the quartile errors.

Figure 2 shows the average size distributions of the non-refractory aerosol mass fractions and *κ*_a_ and *κ*_t_ obtained during this campaign. The chemical compositions of particles with diameters <50 nm were ignored due to the large uncertainty in the AMS measurements. More than half of the chemical compositions obtained at LinAn were inorganic when particles >150 nm; the inorganic fraction decreased to <20% in particles <52 nm in diameter. The measured effective hygroscopicity parameters *κ*_a_ and *κ*_t_ were plotted with their corresponding activation diameters and the scale of the *κ*-axis (the right-hand side of Fig. 2) was chosen to match the scale of the mass fraction based on the expected relationship between the hygroscopicity and chemical composition. The values of *κ*_org_ and *κ*_inorg_ were determined to be 0.2 and 0.6, respectively, assuming that all the particles were either organic or inorganic. The fitting result of *f*_*org*_ with *κ*, as well as the uncertainties of *κ*_org_ and *κ*_inorg_ can be found in the supplement. The value of *κ*_inorg_ was consistent with previously reported values^18,33,40,42^, whereas *κ*_org_ was slightly higher than the typical value of 0.1. However, Meng, Yeung^42^ performed the CCN measurement in Hong Kong, and found in haze episodes, the *κ*_org_ ~0.2 gave a better agreement of the relationship between effective hygroscopicity *κ* and organic mass fraction measured by AMS, and Shantz, Chang^43^ also found that predicted CCN concentration were more reliable when assuming *κ*_org_ ~0.2. Li, Lee^44^ found the hazy period exhibited a higher O:C ratio of 0.51, compared to 0.43 and 0.39 under foggy and clear conditions, leading to a higher hygroscopicity of organic aerosols^45–48^. The high value of *κ*_org_ in LinAn may result from the frequent occurrence of haze pollution. The characteristic range for individual organic compounds going from zero for insoluble species to ~0.3 for small soluble molecules such as oxalic acid^20,49^, the *κ*_org_ value obtained in this manuscript is within this range. Figure 2 also shows that the value of *κ*_t_ is more consistent with the relation between the inorganic and organic aerosol fractions, which is similar to the results found at Guangzhou^11^, indicating that both the active and inactive particles in CCN should be taken into account in predictions.

#### CCN prediction using different methods

The CCN prediction was based on the *κ*-Kӧhler function, where *κ* was calculated using the bulk or size-resolved particles chemical composition measured by AMS. Particles were assumed to be either internally or externally mixed. The following four assumptions were therefore used in the calculation of *κ*.

- ***Bulk chemical composition and internal mixing (BI)***. The chemical composition of particles was assumed to be independent of size and the particles were assumed to be internally mixed, i.e., all the particles over the entire size range had identical chemical compositions. The average mass concentration of bulk non-refractory aerosol species measured during a whole supersaturation cycle by AMS were used in Eq. 2 to calculate *κ*.
- ***Bulk chemical composition and external mixing (BE)***. The chemical composition of the particles was assumed to be independent of size and the particles were assumed to be externally mixed over the entire size range, i.e., there were two types of particle of each size (organic and inorganic aerosols) and the concentrations of these two types of particles were identical at each size.
- ***Size-resolved chemical composition and internal mixing (SI)***. The chemical compositions of the aerosol particles were considered to vary over the entire size range and, at each particle size, the particles were assumed to be internally mixed with identical compositions.
- ***Size-resolved chemical composition and external mixing (SE)***. The particle chemical compositions were considered to vary over the entire size range; however, the particles were assumed to be externally mixed in this method (the external mixing of inorganic and organic particles). There were two types of aerosol particles at each particle size: inorganic and organic.

**Figure 3:**
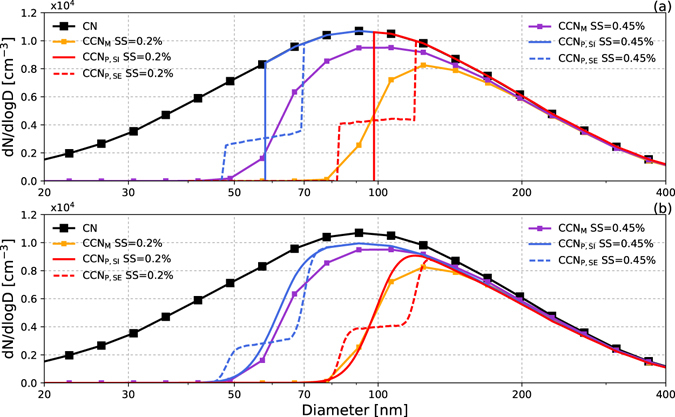
Average CN, CCN number size distribution measured during the campaign and number size distribution of CCN predicted by (**a**) method 1 and (**b**) method 2. The black line is the campaign average CN number distribution; the yellow and purple lines are the measured averaged CCN number size distribution at 0.2 and 0.45% levels of supersaturation, respectively. The red and blue lines are the average predicted CCN number size distribution at 0.2 and 0.45% levels of supersaturation and the solid and dashed lines represent predictions with assumptions of internal and external mixing, respectively. The diamond data points are the median values of all measured spectra.

With these assumptions above, the CCN activation critical diameters (*D*_*c*_) for different SS and *κ* were obtained from the *κ*-Kӧhler function. In the traditional method (method 1), the CCN number concentrations were calculated by integrating the CN number concentration above the critical diameter (*D*_c_) (Fig. 3a). Although this method can successfully predict the total concentration of CCN, it was unable to predict the CCN concentrations for small diameter particles, which may lead to a high level of uncertainty under particular circumstances, such as NPF or a larger amount of freshly emitted fine particles. The CCN number size distribution can be obtained by multiplying the CCN efficiency distribution with CN distribution, thus the CCN efficiency distribution (or the CCN activation fraction function) is essential for this calculation. Su *et al*.^50^ presented a fractional CDF of hygroscopicity *H*(*κ, D*) as shown in Eq. 3, which defined as the number fraction of particles having a hygroscopicity parameter smaller than *κ*. The CCN efficiency distribution represents the fraction of particles activated at a given SS having the effective hygroscopicity parameter equal to or larger than the critical value *κ*_*c*_. Thus, *H*(*κ, D*) is complementary to the CCN efficiency distribution, and the CCN number size distribution can be calculated as Eq. 4:

3 $$H(\kappa ,D)=\sum _{i=1}^{m}{a}_{i}(D)(\frac{1}{2}+\frac{1}{2}{\rm{erf}}(\frac{log\kappa -log{\kappa }_{g,i}(D)}{\sqrt{2}log{\sigma }_{g,i}(D)}))$$

4 $${N}_{ccn}=\int {N}_{d}(1-H(\kappa ,D)dD$$

here *H(κ, D)* can be regarded as the accumulation of multi-mode lognormal CDF at dry diameter *D*, *erf* is the Gauss error function, *a*_*i*_(*D*), *κ*_*g,i*_(*D*) and *σ*_*g,i*_(*D*) are fitting parameters of each mode, representing the number fraction of mode *i*, the geometric mean value of *κ*, and the geometric standard deviation of *κ* in mode *i*, respectively. *N*_*d*_ in Eq. 4 is the number size distribution of CN.

Since 1-*H*(*κ, D*) is the CCN efficiency distribution, we simplify the calculation by directly using the CCN efficiency distribution to calculate the CCN number size distribution. The CDF function (Eq. 5 in Methods) were employed by many studies in CCN efficiency fitting^32,33,36^, thus we use the CDF to estimate the CCN activation efficiency distribution, and multiple it with CN distribution (name method 2 in this paper) to obtain the CCN number size distribution, as well as for total CCN prediction. As analyzed in Methods section, the ratio of fitting standard deviation *σ* and critical diameter *D*_*c*_ represent the aerosol mixing heterogeneity, then we use a single parameter to represent the homogeneity of particles, $\chi =\sigma /{D}_{c}$, and the function can be written as bellow:

5 $${N}_{ccn}=\int a(1+erf\,(\frac{D-{D}_{c}}{\sqrt{2}\chi {D}_{c}})){N}_{d}dD$$

where *D*_*c*_ is the critical diameter at a given SS, *a* is the half of *MAF*_f_. When *κ* > 0.2, an approximate expression, described by Petters and Kreidenweis^20^ (Eq. 10 in their paper) can be used to calculate *κ* and Eq. 5 can be written as:

6 $${N}_{ccn}=\int a{N}_{d}(1+erf((\frac{1}{\sqrt{2}\chi })(\frac{D}{\sqrt[3]{4{A}^{3}/27\kappa l{n}^{2}{S}_{c}}}-1))\,dD$$

where $A=\frac{4{\sigma }_{sol}{M}_{\omega }}{RT{\rho }_{\omega }}$. If the particles are homogenous and all particles with diameters larger than *D*_*c*_ can be activated, i.e., *χ* = 0 and *a* = 0.5, then method 2 is as the same as method 1. The number size distributions of CCN predicted from Eq. 5 are shown in Fig. 3b; the mean values of *a* and *χ* (Table 1) obtained during the campaign were used in the calculation.

Figure 3 shows that the measured CN and CCN number size distributions have the same shape. However, the CCN distribution predicted using method 1 (Fig. 3a) shows the ideal shape, with no CCN for particles smaller than *D*_*c*_ and all particles larger than *D*_*c*_ were activated to droplets. This leads to a larger error between the measured and predicted CCN concentrations around *D*_c_ and is unreliable for CCN predictions at small particle diameters. The size distribution of CCN calculated by method 2 (Fig. 3b), however, was more similar to the measured distribution.

The difference in the shape of CCN distributions predicted by the two methods mainly resulted from the value of *χ*. When *χ* = 0, the CCN concentration increased abruptly at *D*_c_ (Fig. 3a), whereas the CCN concentration increased gradually around *D*_c_ when *χ* > 0, and the larger the value of *χ*, the slower the increase, i.e., the shape of the CCN size distribution was flatter when the particles were more externally and heterogeneously mixed. Note that as a result of instrumental non-idealities, such as heterogeneities in the water vapor supersaturation profile in the CCN counter, or other factors such as the particle shape effect or the differential mobility analyzer transfer function, even the value of *χ* for analytically pure ammonium sulfate particles was as large as 0.03. Thus, the main reason for this gradual rather than abrupt increase in the measured CCN concentration around *D*_c_ was the heterogeneity of particles, while the defects in instruments also take a small part.

In Fig. 3a, *MAF*_f_ = 1, i.e., all the particles were activated to cloud droplets when their diameters were larger than *D*_*c*_, which resulted in an overestimation of the concentrations of CCN. In Fig. 3b, the prediction was much closer to the measured values because the parameter *a* (or *MAF*_f_) was taken into consideration, indicating the existence of less hygroscopic or hydrophobic particles with diameters larger than *D*_c_. However, the addition of *a* may have underestimated the concentration of coarse CCN because it ignored the effect of the increase in diameter on the activation of CCN, i.e., it ignored the fact that all the particles should have been activated when they were large enough. To reduce this underestimation, assumptions could be made that all the particles can be activated when their diameters are larger than a certain value. In this work, we calculated the critical diameter of particles with *κ* = 0.05, then assumed that all the particles could be activated when their diameters were larger than this value.

The predicted distribution of CCN using the external mixing assumption showed two steps before all the CCN were activated. Because the particles were identified as two homogeneous types in the external mixing assumption—inorganic and organic particles—these two steps corresponded to the activation of inorganic and organic particles and the small increase in the concentrations of CCN between the two steps resulted from the increase in the inorganic fraction. Figure 3b shows that the distribution of CCN predicted by method 2 with the assumption of internal mixing was closer to the measured results because the parameter *χ* contains information on the mixing status. When the value of *D*_c_ was calculated with the extreme assumption of total internal mixing, the CCN predicted by method 2 were not completely mixed internally.

#### Comparison of prediction results

**Figure 4:**
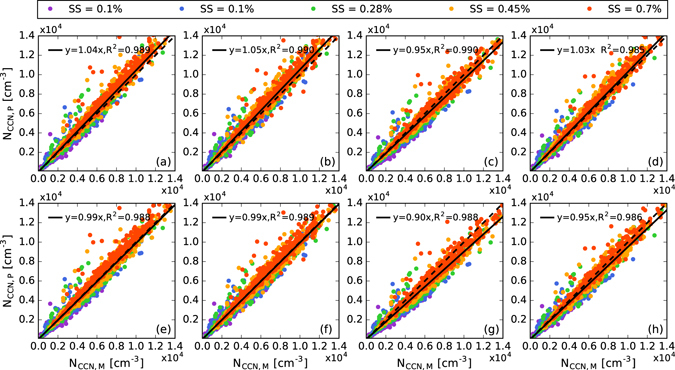
Correlation of measured and predicted concentrations of CCN by (**a**,**e**) BI, (**b**,**f**) SI, (**c**,**g**) BE and (**d**,**h**) SE assumptions, respectively. The predicted CCN concentration in (**a**–**d**) were calculated by method 1 and those in (**e**–**h**) were calculated by method 2. The colored dots are the data values under different supersaturations; the solid lines are least-squares fits to all the data and the dashed lines are the 1:1 lines.

The predicted CCN concentration (*N*_CCN, P_) was fitted with the observed concentration of CCN (*N*_CCN, M_) by orthogonal distance regression and weighted by 5% uncertainties in both measurement and prediction. The fitting results are shown in Fig. 4.

The predictions in Fig. 4a,b,e and f were based on the internal mixing assumption, for all levels of supersaturation, and the total concentrations of CCN calculated by method 2 were closer to the measured values. The slope of fitting lines for each level of supersaturation are given in Table 2. Method 2 with BI and SI assumptions gave better results for *SS* ≥ 0.28%, indicating that in environments with high levels of supersaturation, such as convection clouds, the concentrations of CCN predicted by method 2 were more accurate, even for the total concentration of CCN. However, when *SS* = 0.1%, method 1 with the assumption of internal mixing showed better results and method 2 slightly underestimated the concentration of CCN as a result of the underestimation of the concentration of large diameter CCN; this was aggravated by the low value of *a*. This underestimation could be reduced by assuming that all the particles can be activated when their diameters are larger than a certain value.

**Table 2:** The slope of fitting lines for the measured and predicted concentrations of CCN by two different methods.

SS	BI		SI		BE		SE	
	Method1	Method2	Method1	Method2	Method1	Method2	Method1	Method2
**0.1%**	0.97	0.92	1.00	0.97	0.89	0.84	0.97	0.89
**0.2%**	1.03	0.97	1.02	0.98	0.94	0.88	0.99	0.91
**0.28%**	1.06	0.98	1.04	0.98	0.96	0.89	1.01	0.93
**0.45%**	1.08	1.01	1.07	1.00	0.99	0.92	1.04	0.96
**0.7%**	1.08	1.06	1.08	1.03	1.00	0.97	1.05	1.00

For predictions with the assumption of external mixing, method 1 was always better than method 2 regardless of the size distribution of the chemical composition of the aerosols (Fig. 4c,d,g and h and Table 2). This was because method 2 already contained external mixing information and the repeated assumption of external mixing would overestimate this. In general, the predictions using method 2 with the assumption of internal mixing were the best; for each level of supersaturation, method 2 with the SI assumption were the best, indicating the importance of information about the size-resolved chemical composition and the degree of particle homogeneity for the accurate prediction of CCN.

#### Analysis of prediction errors

**Figure 5:**
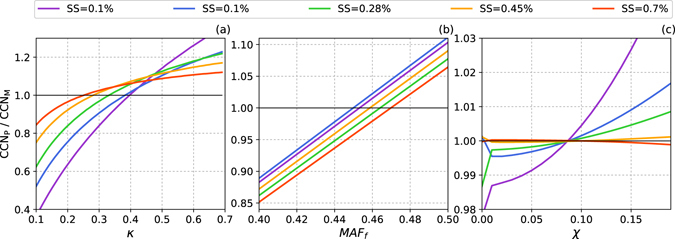
Prediction errors for CCN caused by variations in the prediction parameters: (**a**) the hygroscopic parameter *κ*, (**b**) the degree of homogeneity *χ*, and (**c**) half of the maximum activated fraction *a*.

The influence of three parameters—the hygroscopic parameter *κ*, half of the maximum activated fraction *a*, and the degree of homogeneity *χ*—on the prediction of CCN was investigated in this section. Figure 5 shows that the largest error was caused by the hygroscopic parameter *κ*. The ratio of the predicted CCN to measured CCN ranged from about 0.4 to 1.4 (Fig. 5a) and the ratio increased with decreasing levels of supersaturation, indicating that the prediction of CCN was more sensitive to particle hygroscopicity at low levels of supersaturation. The errors caused by the underestimation of *κ* were higher than those caused by its overestimation. This further illustrated the importance of water-soluble organic particles because these were dominant at small diameters and neglecting this size range would lead to an underestimation of *κ*.

The parameters *a* and *χ*, which can lead to prediction errors of about 15 and 3%, respectively (Fig. 5b,c), have less impact on the predicted total concentration of CCN. Thus for the prediction of the total concentration of CCN, the homogeneous parameter, and sometimes also *MAF*_f_, could be ignored in the simplified calculation. The major difference between methods 1 and 2 was caused by *MAF*_f_ (or *a*) because the prediction error caused by *χ* can be neglected.

**Figure 6:**
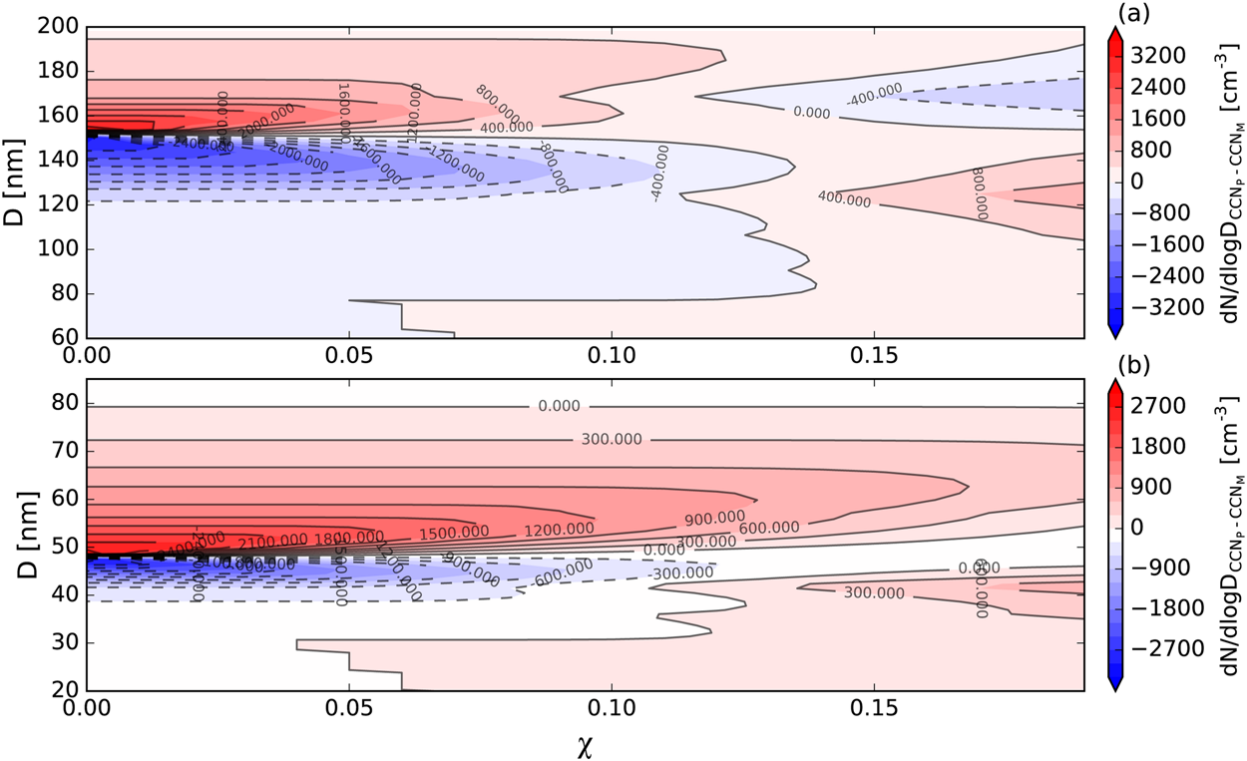
Size-resolved CCN prediction error caused by variations in the heterogeneity parameter *χ* at (**a**) 0.1% and (**b**) 0.7% supersaturation. The color bar represents the prediction error, which was calculated as the number distribution (d*N*/dlog*D*) of the predicted CCN (*CCN*_P_) subtracted the measured CCN (*CCN*_M_).

However, an incorrect value of *χ* can lead to a high prediction error in CCN at different values of *D*_c_ (Fig. 6). A prediction with a lower value of *χ* would underestimate the concentration of CCN below *D*_c_ and overestimate it above *D*_c_. As the underestimation and overestimation can compensate for each other, the total CCN concentration shows a much smaller prediction error (Fig. 5c).

However, under some circumstances in which the aerosol size distribution peaks at small diameters (<150 nm), such as in NPF events, the error is not completely compensated for and the number concentration of small diameter CCN particles is needed to give an accurate prediction.

A comparison of Fig. 6a,b shows that *χ* caused a larger prediction error around *D*_c_ at 0.1% supersaturation, which may because the *D*_c_ value at this supersaturation approximated the mode diameter of the aerosol accumulation mode where the CN concentration was high. Figure 6b shows that when *SS* = 0.7%, the overestimated concentration of CCN was much higher than the underestimation at low values of *χ*, which only occurred in the diameter range of about 40–49 nm; while overestimation occurred over larger ranges of diameter from about 49 to 80 nm. This overestimation may significantly influence the prediction of the CCN concentration at the Aitiken mode, especially during NPF events; it may also overestimate the total concentration of CCN.

#### Summary of prediction parameters

**Figure 7:**
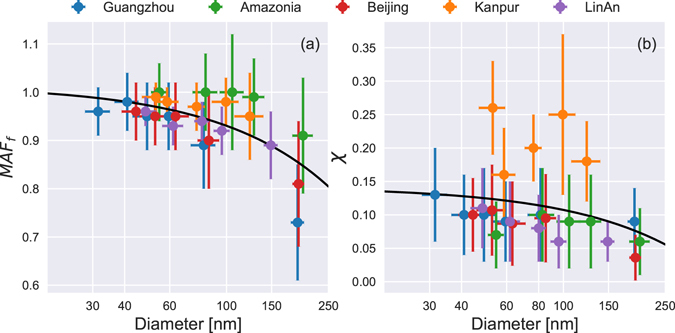
Measured values of (**a**) *MAF*_f_ and (**b**) *χ* plotted against the critical diameters of aerosol particles. The points are the mean values of the measurements and the error bars represent the standard deviations. The solid black line is the fitting line. The blue data are from Rose, Nowak^11^, the green data are from Gunthe, King^33^, the red data are from Gunthe, Rose^36^, the orange data are from Bhattu and Tripathi^35^, and the violet data are from the research reported here.

Despite the complexity of organic aerosols, the values of *κ* for different aerosol species, especially inorganic and some organic particles, have already been investigated under laboratory conditions. The values of *χ* and *MAF*_f_ are summarized in Fig. 7, which includes data from previously published papers as well as the research reported here.

The value of *MAF*_f_, or 2*a*, ranged from 1.0 to 0.8 (Fig. 7a). A higher level of supersaturation corresponds to smaller values of *D*_c_ and higher values of *MAF*_f_, which made *a* approximately equal to 0.5; at lower level of supersaturation, the value of *a* was about 0.4. As discussed earlier in this paper, a low value of *a* in the prediction of CCN at low levels of supersaturation will cause an error because it neglects the fact that particles can usually all be activated when they are large enough and therefore assumptions are needed. The relationship between the measured values of *MAF*_f_ and the diameter of particles was fitted by the following equation: *MAF*_f_ = 1.01−8.4/10,000*D*. As a result of the large variation in the chemical components of aerosols at different locations, their actual *MAF*_f_ values may deviate slightly from the fitted values, as seen in the Amazon region, and the fraction of small inactive particles such as soot may result in much higher *MAF*_f_ values than in urban areas.

Figure 7b shows that the value of *χ* in Kanpur was higher than in other areas, which may result from the frequent occurrence of dust events in the sampling location because mineral aerosols can increase the heterogeneity of particles around *D*_c_, leading to a higher value for *χ*. The variation in *χ* was generally in the range 0.05–0.15 and its relationship with diameter was fitted as *χ* = 0.14−3.4/10,000*D*. Although the fitting results for *χ* had a larger error than those for *MAF*_f_, *χ* only caused a small prediction error when it was overestimated (Fig. 6), i.e., as long as *χ* was larger than a certain value (0.1 in Fig. 7), then the prediction error around the critical diameter was negligible.

## Discussion

An experiment was carried out in 2013 at LinAn, a regional GAW station, to investigate the long-term activation characteristics of CCN and obtain accurate size-resolved number concentration predictions of CCNs in the YRD.

The average values of *MAF*_f_ and the critical active diameters of aerosol particles at LinAn varied from about 0.89 to 0.96 and about 150 to 45 nm for levels of supersaturation ranging from 0.1 to 0.7%, respectively. The corresponding value of *κ* varied from about 0.4 to 0.26. The CCN at LinAn, rural station but within area of high rapid economic development and population density of China, were more easily activated than in clean (e.g., the Amazon region) or dusty (e.g., Kanpur-spring) areas, but were harder to activate than in more polluted areas (e.g., Beijing). The fraction of inactive particles at LinAn was similar to that in clean areas (the Amazon region), but was smaller than in more polluted areas (Beijing). The degree of heterogeneity at LinAn decreased with an increase in the critical active diameter of aerosol particles, indicating that larger particles were usually more homogenous. For the same critical active diameter, the degree of heterogeneity at LinAn was similar to that in the Amazon region, but was smaller than at Kanpur-spring and Guangzhou within another area of high rapid economic development and population density of China, but with less pollution, indicating that the aerosols at LinAn were more aged and more homogeneous than in more polluted areas.

The CCN were predicted by the *κ*-Kӧhler function using the measured aerosol number size distribution and the chemical composition, where *κ* was calculated from the mass weighted average using AMS measurements. The *κ* values of inorganic and organic particles were 0.6 and 0.2, respectively. An improved method for calculating CCN was used here with two additional parameters (*a* representing half the value of *MAF*_f_ and *χ* for the degree of heterogeneity). The traditional prediction method (called method 1) was unable to predict the size-resolved CCN and resulted in large errors in the concentrations of CCN around *D*_c_, while the size distributions of CCN predicted by the improved method (called method 2) were a better fit to the measured data. The total concentration of CCN predicted with method 2 and the SI assumption showed the best agreement with the measured data.

The difference in the CCN distributions predicted with methods 1 and 2 was mainly caused by *χ*, which depends mainly on the heterogeneity of the particles. However, the prediction error analysis showed that *χ* had little influence on the total concentration of CCN. This is because the CCN concentration is underestimated for particles smaller than *D*_c_ and overestimated for particles larger than *D*_c_ with incorrect *χ*. The extent of the over- and underestimation was generally the same and thus the effect could be balanced. However, for predictions of the CCN concentration at small diameters, *χ* is important because the under- and overestimation are not be the same if a large number of particles have small diameters ( < 150 nm), such as during an NPF event. The prediction error contributed from *MAF*_f_ to the total concentration of CCN was as large as 15%. The size distribution of CCN calculated with *MAF*_f_ (*a*) was closer to the measured data; however, *MAF*_f_ may neglect the effects of particle diameter on CCN activation, especially at low levels of supersaturation (corresponding to larger particles). Method 2 is most sensitive to *κ*, especially at low levels of supersaturation. Underestimating *κ* will lead to a much larger error than overestimating *κ*, indicating the importance of small, water-soluble organic aerosols. Previously reported values of *MAF*_f_ and *χ* were also reviewed and empirical equations describing their variation with diameter were proposed.

## Methods

### Field station and experimental setup

The characteristics of CCN and other aerosol properties—such as the particle size distribution and chemical composition—were measured at the LinAn regional GAW station in the YRD, China from 1 January to 30 October 2013. Detailed information on the site and surroundings have been reported previously by Zhang, Sun^51^.

An aerosol inlet system based on a commercially available PM_10_ impactor (PM_10_ Inlet, URG Corporation) was installed on a rooftop (*c*. 5 m a.g.l.). The aerosols were dried to < 30% relative humidity by an automatically regenerating adsorption aerosol dryer^52^. The following instruments were then used to make simultaneous measurements: an Aerodyne aerosol mass spectrometer to determine the chemical composition of the fine particles^23^; a twin differential mobility particle sizer to determine the particle number size distribution^31^; and a size-resolved CCN measurement system including a differential mobility analyzer (Model 3080, TSI), a condensation particle counter (Model 3772, TSI) and a continuous-flow CCN counter (Model CCN-100, DMT) to determine the size-resolved CCN activation properties^53^.

The CCN counter was operated at five levels of supersaturation: 0.1, 0.2, 0.28, 0.45, and 0.7%. At each level of supersaturation, 12 set diameters of dry particles ranging from 20 to 300 nm were selected by the differential mobility analyzer and then the total number concentration of aerosol particles (condensation nuclei, CN) and CCN were measured by the downstream condensation particle and CCN counters. The whole measurement cycle took 3 h; more information about the CCN measurement setting have been reported by Che, Zhang^12^.

### Data processing

Before measurement, the CCN counter was calibrated as described by Rose, Gunthe^32^ and three further calibrations were carried out during the measurements. The CCN data were corrected for multiple charges and the differential mobility analyzer transfer function as described by Rose, Gunthe^32^ and Frank, Dusek^54^. After correction, the measured CCN efficiency spectra were fitted by the following three- and two-parameter cumulative Gaussian distribution functions (CDFs), respectively^32^:

7 $${f}_{{N}_{ccn}/{N}_{cn}}=a(1+erf\,(\frac{D-{D}_{a}}{{\sigma }_{a}\sqrt{2}}))$$

8 $${f}_{{N}_{ccn}/{N}_{cn}}=0.5(1+erf\,(\frac{D-{D}_{t}}{{\sigma }_{t}\sqrt{2}}))$$

For the three-parameter CDF (Eq. 7), *a* is the half-maximum activation fraction, i.e., *a* = 0.5 for an ideal completely activated aerosol particle. Therefore, *a* is forced to be equal to 0.5 by assuming that all the particles are activated at the measured maximum diameter in the two-parameter CDF (Eq. 8). *D*_a_ and *D*_t_ represent the critical active diameters determined from the three-parameter and two-parameter CDFs; *σ*_a_ and *σ*_t_ are the standard deviations for two CDFs. The ratio of *σ*_a_ and *σ*_t_ to their corresponding diameters can be regarded as an indicator of the degree of heterogeneity of the aerosol particle. *σ*_a_/*D*_a_ represents the heterogeneity of the CCN active particle fraction around *D*_a_; *σ*_t_/*D*_t_ represents the overall heterogeneity of the active and inactive portions of CCN around *D*_t_. Detailed information about the CDFs has been reported by Rose, Nowak^11^.

The measured effective hygroscopicity parameter, *κ*, was calculated by the following equation^20,32^:

9 $$SS=\frac{{D}_{drop}^{3}-{D}^{3}}{{D}_{drop}^{3}-{D}^{3}(1-\kappa )}\exp (\frac{4{\sigma }_{sol}{M}_{\omega }}{RT{\rho }_{\omega }{D}_{drop}})$$

where *SS* is the equilibrium supersaturation, *D*_drop_ is the droplet diameter, *M*_ω_ is the molecular weight of water, *D* is the dry particle diameter, *R* is the gas constant, *T* is the absolute temperature, and *ρ*_ω_ is the density of water. The values of *κ*_a_ and *κ*_t_ were obtained by inserting the setting supersaturation and the observed critical diameters (*D*_a_ and *D*_t_), and can be used to represent the average hygroscopicity of active CCN particles at about *D*_a_ and all particles (CCN active and inactive) at about *D*_t_, respectively.

## Electronic supplementary material

Supplementary Information

## References

[1] Boucher, O. *et al*. Clouds and Aerosols. In: Stocker, T. F. *et al*. (eds). Climate Change 2013*:* The Physical Science Basis. Contribution of Working Group I to the Fifth Assessment Report of the Intergovernmental Panel on Climate Change. Cambridge University Press: Cambridge, United Kingdom and New York, NY, USA, pp 571–658 (2013).

[2] Rotstayn, L. D. Indirect forcing by anthropogenic aerosols: A global climate model calculation of the effective‐radius and cloud‐lifetime effects. Journal of Geophysical Research: Atmospheres (1984–2012), **104**(D8): 9369–9380 (1999).

[3] Rosenfeld D (2008). Flood or drought: how do aerosols affect precipitation?. science.

[4] Ramanathan V, Crutzen P, Kiehl J, Rosenfeld D (2001). Aerosols, climate, and the hydrological cycle. science.

[5] Pierce JR, Adams PJ (2009). Uncertainty in global CCN concentrations from uncertain aerosol nucleation and primary emission rates. Atmospheric Chemistry And Physics.

[6] Andreae MO, Rosenfeld D (2008). Aerosol-cloud-precipitation interactions. Part 1. The nature and sources of cloud-active aerosols. Earth-Science Reviews.

[7] Dusek U (2006). Size matters more than chemistry for cloud-nucleating ability of aerosol particles. Science.

[8] Ervens B (2007). Prediction of cloud condensation nucleus number concentration using measurements of aerosol size distributions and composition and light scattering enhancement due to humidity. Journal of Geophysical Research.

[9] Ovadnevaite J (2011). Primary marine organic aerosol: A dichotomy of low hygroscopicity and high CCN activity. Geophysical Research Letters.

[10] Orellana MV (2011). Marine microgels as a source of cloud condensation nuclei in the high Arctic. Proceedings of the National Academy of Sciences of the United States of America.

[11] Rose D (2010). Cloud condensation nuclei in polluted air and biomass burning smoke near the mega-city Guangzhou, China–Part 1: Size-resolved measurements and implications for the modeling of aerosol particle hygroscopicity and CCN activity. Atmospheric Chemistry and Physics.

[12] Che HC (2016). Characterization and parameterization of aerosol cloud condensation nuclei activation under different pollution conditions. Scientific Reports.

[13] Wang J, Cubison M, Aiken A, Jimenez J, Collins D (2010). The importance of aerosol mixing state and size-resolved composition on CCN concentration and the variation of the importance with atmospheric aging of aerosols. Atmospheric Chemistry and Physics.

[14] Paramonov M (2015). A synthesis of cloud condensation nuclei counter (CCNC) measurements within the EUCAARI network. Atmospheric Chemistry and Physics.

[15] Deng Z (2013). An examination of parameterizations for the CCN number concentration based on *in situ* measurements of aerosol activation properties in the North China Plain. Atmospheric Chemistry and Physics.

[16] Ervens B (2010). CCN predictions using simplified assumptions of organic aerosol composition and mixing state: a synthesis from six different locations. Atmospheric Chemistry and Physics.

[17] Bhattu D, Tripathi S (2015). CCN closure study: Effects of aerosol chemical composition and mixing state. Journal of Geophysical Research: Atmospheres.

[18] Jurányi Z (2010). Measured and modelled cloud condensation nuclei number concentration at the high alpine site Jungfraujoch. Atmospheric Chemistry and Physics.

[19] Deng Z (2011). Size-resolved and bulk activation properties of aerosols in the North China Plain. Atmospheric Chemistry and Physics.

[20] Petters M, Kreidenweis S (2007). A single parameter representation of hygroscopic growth and cloud condensation nucleus activity. Atmospheric Chemistry and Physics.

[21] Petters M, Kreidenweis S (2008). A single parameter representation of hygroscopic growth and cloud condensation nucleus activity–Part 2: Including solubility. Atmospheric Chemistry and Physics.

[22] Kulmala M (2004). Formation and growth rates of ultrafine atmospheric particles: a review of observations. Journal of Aerosol Science.

[23] Zhang Y (2015). Significant concentration changes of chemical components of PM 1 in the Yangtze River Delta area of China and the implications for the formation mechanism of heavy haze–fog pollution. Science of The Total Environment.

[24] Mao KB (2014). Global aerosol change in the last decade: An analysis based on MODIS data. Atmospheric Environment.

[25] Zhang X (2012). Atmospheric aerosol compositions in China: spatial/temporal variability, chemical signature, regional haze distribution and comparisons with global aerosols. Atmospheric Chemistry and Physics.

[26] Zhang Q (2012). Impact of aerosol composition on cloud condensation nuclei activity. Atmospheric Chemistry and Physics.

[27] Zhang F (2014). Aerosol hygroscopicity and cloud condensation nuclei activity during the AC3Exp campaign: implications for cloud condensation nuclei parameterization. Atmos Chem Phys.

[28] Ma N *et al*. Variation of CCN activity during new particle formation events in the North China Plain. Atmospheric Chemistry & Physics 1–25 (2016).

[29] Zhang XY (2012). Atmospheric aerosol compositions in China: spatial/temporal variability, chemical signature, regional haze distribution and comparisons with global aerosols. Atmos Chem Phys.

[30] Huang R (2014). High secondary aerosol contribution to particulate pollution during haze events in China. Nature.

[31] Shen X (2015). Characterization of submicron aerosols and effect on visibility during a severe haze-fog episode in Yangtze River Delta, China. Atmospheric Environment.

[32] Rose D (2008). Calibration and measurement uncertainties of a continuous-flow cloud condensation nuclei counter (DMT-CCNC): CCN activation of ammonium sulfate and sodium chloride aerosol particles in theory and experiment. Atmospheric Chemistry and Physics.

[33] Gunthe S (2009). Cloud condensation nuclei in pristine tropical rainforest air of Amazonia: size-resolved measurements and modeling of atmospheric aerosol composition and CCN activity. Atmospheric Chemistry and Physics.

[34] Cubison M (2008). The influence of chemical composition and mixing state of Los Angeles urban aerosol on CCN number and cloud properties. Atmospheric Chemistry and Physics.

[35] Bhattu D, Tripathi SN (2014). Inter-seasonal variability in size-resolved CCN properties at Kanpur, India. Atmospheric Environment.

[36] Gunthe S (2011). Cloud condensation nuclei (CCN) from fresh and aged air pollution in the megacity region of Beijing. Atmospheric Chemistry and Physics.

[37] Kuwata M (2008). Cloud condensation nuclei activity at Jeju Island, Korea in spring 2005. Atmospheric Chemistry and Physics.

[38] Adhikari, M. *et al*. Vertical distribution of cloud condensation nuclei concentrations and their effect on microphysical properties of clouds over the sea near the southwest islands of Japan. Journal of Geophysical Research: Atmospheres (1984–2012) **110**(D10) (2005).

[39] Lance, S. *et al*. Cloud condensation nuclei activity, closure, and droplet growth kinetics of Houston aerosol during the Gulf of Mexico Atmospheric Composition and Climate Study (GoMACCS). Journal of Geophysical Research: Atmospheres (1984–2012), **114**(D7) (2009).

[40] Rose D (2011). Cloud condensation nuclei in polluted air and biomass burning smoke near the mega-city Guangzhou, China–Part 2: Size-resolved aerosol chemical composition, diurnal cycles, and externally mixed weakly CCN-active soot particles. Atmospheric Chemistry and Physics.

[41] Cross ES (2007). Laboratory and ambient particle density determinations using light scattering in conjunction with aerosol mass spectrometry. Aerosol Science and Technology.

[42] Meng J, Yeung M, Li Y, Lee B, Chan C (2014). Size-resolved cloud condensation nuclei (CCN) activity and closure analysis at the HKUST Supersite in Hong Kong. Atmospheric Chemistry and Physics.

[43] Shantz NC (2010). Slower CCN growth kinetics of anthropogenic aerosol compared to biogenic aerosol observed at a rural site. Atmos Chem Phys.

[44] Li YJ, Lee BYL, Yu JZ, Ng NL, Chan CK (2013). Evaluating the degree of oxygenation of organic aerosol during foggy and hazy days in Hong Kong using high-resolution time-of-flight aerosol mass spectrometry (HR-ToF-AMS). Atmos Chem Phys.

[45] Lambe A (2011). Laboratory studies of the chemical composition and cloud condensation nuclei (CCN) activity of secondary organic aerosol (SOA) and oxidized primary organic aerosol (OPOA). Atmospheric Chemistry and Physics.

[46] Massoli, P. *et al*. Relationship between aerosol oxidation level and hygroscopic properties of laboratory generated secondary organic aerosol (SOA) particles. Geophysical Research Letters **37**(24) 2010.

[47] Mei F, Setyan A, Zhang Q, Wang J (2013). CCN activity of organic aerosols observed downwind of urban emissions during CARES. Atmospheric Chemistry and Physics.

[48] Moore, R. *et al*. Hygroscopicity and composition of California CCN during summer 2010. Journal of Geophysical Research: Atmospheres **117**(D21) (2012).

[49] Mikhailov E, Vlasenko S, Martin ST, Koop T, Pöschl U (2009). Amorphous and crystalline aerosol particles interacting with water vapor: conceptual framework and experimental evidence for restructuring, phase transitions and kinetic limitations. Atmos Chem Phys.

[50] Su H (2010). Hygroscopicity distribution concept for measurement data analysis and modeling of aerosol particle mixing state with regard to hygroscopic growth and CCN activation. Atmospheric Chemistry and Physics.

[51] Zhang L (2015). Observations of relative humidity effects on aerosol light scattering in the Yangtze River Delta of China. Atmospheric Chemistry and Physics.

[52] Tuch TM (2009). Design and performance of an automatic regenerating adsorption aerosol dryer for continuous operation at monitoring sites. Atmos Meas Tech.

[53] Roberts G, Nenes A (2005). A continuous-flow streamwise thermal-gradient CCN chamber for atmospheric measurements. Aerosol Science and Technology.

[54] Frank G, Dusek U, Andreae M (2006). Technical note: A method for measuring size-resolved CCN in the atmosphere. Atmospheric Chemistry and Physics Discussions.

