# Author Correction: Prediction of size-resolved number concentration of cloud condensation nuclei and long-term measurements of their activation characteristics

**DOI:** 10.1038/s41598-018-24985-w

**Published:** 2018-05-23

**Authors:** H. C. Che, X. Y. Zhang, L. Zhang, Y. Q. Wang, Y. M. Zhang, X. J. Shen, Q. L. Ma, J. Y. Sun, J. T. Zhong

**Affiliations:** 1 Key Laboratory of Atmospheric Chemistry of CMA, Institute of Atmospheric Composition, Chinese Academy of Meteorological Sciences, Beijing, 100081 China; 2 College of Earth Science, University of Chinese Academy of Sciences, Beijing, 100049 China; 3 State Key Laboratory of Severe Weather, Chinese Academy of Meteorological Sciences, Beijing, 100081 China; 4 Center for Excellence in Regional Atmospheric Environment, IUN, CAS, Xiamen, 361021 China; 5 LinAn Regional Global Atmosphere Watch Station, LinAn, 311307 China; 6 State Key Laboratory of Cryospheric Sciences, Cold and Arid Region Environmental and Engineering Research Institute, Chinese Academy of Sciences, Lanzhou, 730000 China

Correction to: *Scientific Reports* 10.1038/s41598-017-05998-3, published online 19 July 2017

In the original version of this Article, the Supplementary Information file was omitted. This has now been corrected in the PDF and HTML versions of the Article.

