# Supplementary material for: Prediction of size-resolved number concentration of cloud condensation nuclei and long-term measurements of their activation characteristics
Source: Sci Rep. 2017 Jul 19;7:5819. doi: 10.1038/s41598-017-05998-3 (PMC5517613; doi:10.1038/s41598-017-05998-3)
Supplement: Supplementary file 1 — Supplementary Information [file 41598_2017_5998_MOESM1_ESM.pdf]

Prediction of size-resolved number concentration of cloud condensation nuclei and long-term measurements of their activation characteristics

H. C. Che[1,2], X. Y. Zhang[3,*], L. Zhang[1,2], Y. Q. Wang[1], Y. M. Zhang[1], X. J. Shen[1], Q. L. Ma[4], J. Y. Sun[1,5], J. T. Zhong[1]

[1]Key Laboratory of Atmospheric Chemistry of CMA, Institute of Atmospheric Composition, Chinese Academy of Meteorological Sciences, Beijing 100081, China

[2]College of Earth Science, University of Chinese Academy of Sciences, Beijing 100049, China

[3]Chinese Academy of Meteorological Sciences, Beijing 100081, China & Center for Excellence in Regional Atmospheric Environment, IUN, CAS

[4]LinAn Regional Global Atmosphere Watch Station, LinAn 311307, China

[5]State Key Laboratory of Cryospheric Sciences, Cold and Arid Region Environmental and Engineering Research Institute, Chinese Academy of Sciences, Lanzhou 730000, China

Corresponding: X. Y. Zhang (xiaoye@camscma.cn)

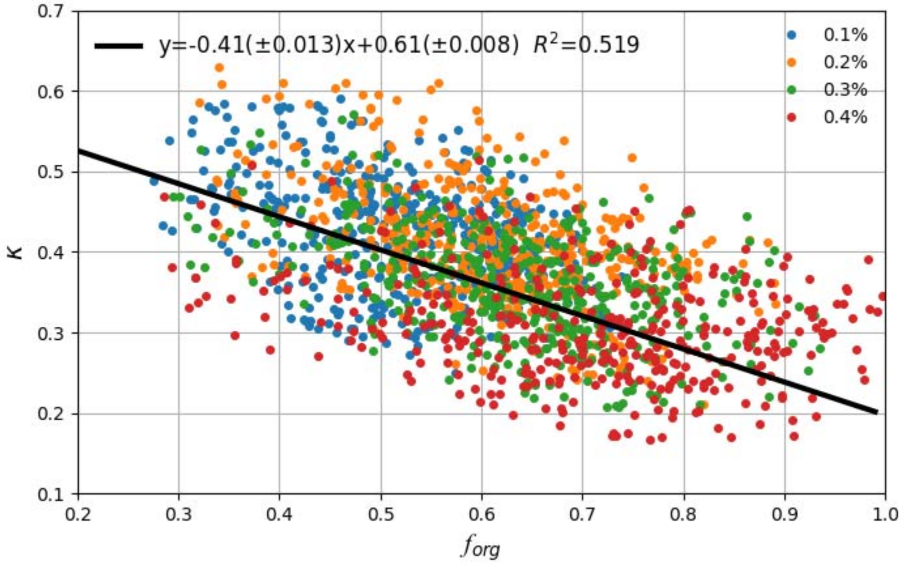

Figure S1. Correlation between the effective hygroscopicity parameter of CCN-active particles (κ_a) observed at four different supersaturations and the organic mass fraction (f_{org}) determined by size-resolved AMS measurements. The data were fitted by orthogonal distance regression with both 10 % relative error for measured κ_a and f_{org}.

As showed in the figure S1, the low correlation coefficient obtained with the size-resolved AMS data ($R^2 = 0.52$) is a result of low signal-to-noise of these data. However, the fitting line generally showed the tendency of the relation of f_{org} with $\kappa_{a.}$ The line fit equation for all data is y = -0.41(±0.013)x+0.61(±0.008), with standard deviations in brackets. Extrapolation of the fit line to x=1 (f_{org}=1) yields an effective hygroscopicity parameter of $\kappa_{org} \approx 0.2 \pm 0.02$, and to x=0 ($f_{org} = 0$) yields an effective hygroscopicity parameter of $\kappa_{inorg} \approx 0.61 \pm 0.008$.
